# Tooth ARcademy: A mobile app for teaching and learning of oral histology

**DOI:** 10.1371/journal.pone.0329172

**Published:** 2025-07-24

**Authors:** Nazlee Sharmin, Hady Abdallah, Elias Jirgees, Ava K. Chow

**Affiliations:** Mike Petryk School of Dentistry, Faculty of Medicine & Dentistry, College of Health Sciences, University of Alberta, Edmonton, Alberta, Canada; King Faisal University, SAUDI ARABIA

## Abstract

m-Learning is gaining popularity in health professional education; however, reports on mobile apps targeting didactic teaching and learning are scarce, particularly in the context of health professional courses such as histology. Histology is an essential foundational component of dental and medical education. At the Mike Petryk School of Dentistry, University of Alberta, instructors utilize photomicrographs from textbooks to teach students on the microanatomy of teeth, the development of tooth and facial regions, and developmental anomalies. Limited availability of high-quality tissue sections and time constraints present challenges for both students and instructors. To provide students with an accessible collection of diverse histological sections and to facilitate in-class interactive didactic teaching, we developed an Augmented Reality (AR)-based mobile app called Tooth ARcademy. The development of Tooth ARcademy comprises the following steps: selecting histology glass slides, digitizing the glass slides, curating and annotating the digital slides, preparing multiple-choice questions, and integrating the resources into the mobile app. Tooth ARcademy is available worldwide at no cost. The app has three modes. Instructors can use the AR-based *Learn* mode to create in-class activities and supplemental questions tailored to students with specific learning outcomes. The *Practice* mode enables students to study oral histology outside of class time. With the *Quiz* mode of Tooth ARcademy, students can self-assess their knowledge of oral histology by participating in quizzes. The knowledge of oral histology is essential for dental education. Tooth ARcademy is designed to create interactive and engaging learning environments both inside and outside the classroom. Besides some limitations of the current phase, Tooth ARcademy can be a valuable m-learning tool that benefits students and educators in dental, medical, and other professional schools.

## Introduction

Mobile devices have become an integral part of our everyday lives. Recent technological advancements have transformed our smartphones into sophisticated computers capable of running complex applications (apps), impacting many aspects of our lives [1]. This widespread use has led to the emergence of mobile-enabled teaching and learning, or m-learning [1].

The development of innovative apps partly triggers the increased use of mobile devices. ‘App’ is an abbreviated form of the word ‘application.’ The increased popularity of mobile-enabled learning (m-learning) is driven by students’ growing use of apps for academic purposes. Mohapatra et al. reported that medicine-specific apps used by medical students include medication guides, clinical handbooks, discipline-specific references, clinical examination apps, simulation apps, medical calculators, and apps with diagnostic abilities, all aimed at improving patient care and clinical skills [2]. The most recommended mobile apps include Capsule, a clinical case-based quiz app; MDCalc Medical Calculator; the Forest App to track productivity of the day; and the Clinical Odyssey simulation app [3]. Similarly, the most recommended and downloaded apps for dental and nursing students are also focused on patient care and clinical skill development, with a few specifically designed for foundational science and didactic teaching [4,5].

The positive impact of m-learning is well-documented for students in health professional education [4,6–8]. However, few mobile apps currently focus on promoting active engagement in classroom-based didactic activities. Didactic learning of basic science concepts is essential to dental and medical education. As mobile apps are shown to improve knowledge acquisition [4,6], it is high time for educators to design and develop apps to enhance student engagement in traditional didactic classroom settings. This paper describes the development of Tooth ARcademy, a mobile app developed to facilitate didactic teaching and learning of oral histology.

Histology, the study of the microscopic structure of cells and tissues, is a cornerstone of medical and dental curricula [9]. This morphofunctional science, which aims to describe the relationship between the form and function of a biological system, is highly dependent on the intensive use of images. Such heavy reliance on high-quality images and photomicrographs poses a challenge for educators to teach and students to comprehend [9,10]. In student perception, unfamiliar terminology, the complexity of the material, insufficient class time, and inadequate photomicrographs make learning histology challenging [9]. A review of the existing literature on the application of digital information and communication technologies on histology learning identified only two studies that apply m-learning in the classroom to teach histology: an online game Kahoot, played with histology questions in a dental school, and NDER: Novel Diagnostic Educational Resource, a web-based software for medical students where an annotated histology image is displayed briefly, asking users to identify the tissue type [10–12].

Histology is an essential foundational content taught extensively in the Doctor of Dental Surgery (DDS) and Dental Hygiene (DH) programs at the Mike Petryk School of Dentistry, University of Alberta. In the didactic classroom, instructors use photomicrographs from textbooks and digital slides to teach students the microanatomy of teeth, the development of tooth and facial regions, and developmental anomalies. As learning outcomes, students are expected to identify the type of cross-section (longitudinal, transverse, or oblique), the method of slide preparation (ground section or demineralized section), the tissue type, the stage of development (where applicable), and the underlying structural components of a given tissue section. To identify a tissue section, students must also apply their knowledge of magnification and perform mental rotation, a set of skills that improve with practice. The bottleneck of traditional teaching is the lack of sufficient and diverse high-quality tissue sections (glass slides and photomicrographs) and insufficient class time.

To facilitate in-class learning and to provide students with an extensive collection of diverse histological sections, we developed an Augmented Reality (AR)-based mobile app called Tooth ARcademy. AR is an emerging interactive technology that incorporates digital data into the real world [13]. When looking through an AR-adaptable device’s display or camera, the user can see their surrounding world with the addition of a digital component that is not physically present in the real world. With Tooth ARcademy, instructors can facilitate in-class activities in an AR-based *Learn* mode, while students can practice and self-assess using *Practice* and *Quiz* modes. Tooth ARcademy is freely available worldwide in the Apple App Store and Google Play Store.

## Materials and methods

### Ethics statement

This article does not involve humans or animals, and thus approval by the relevant institutional review board or ethics committee is not required. No human participants were recruited in this study, and no informed consent was obtained.

### Selection of histology glass slides

**Fig 1:**
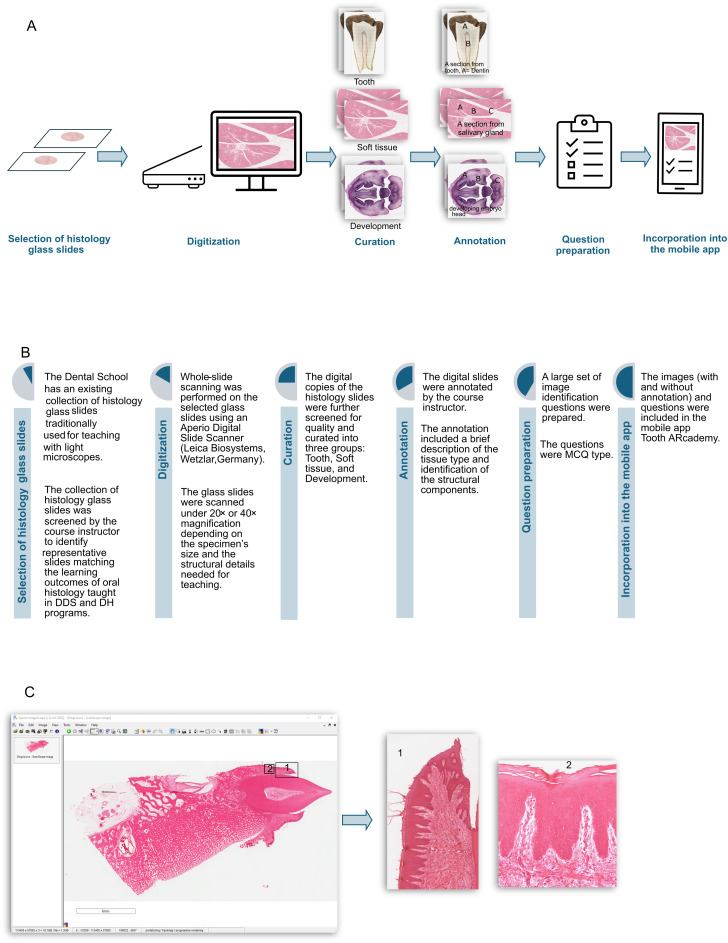
Development of Tooth ARcademy. Tooth ARcademy is a mobile app developed as a teaching and learning resource for oral histology. **(A, B)** Steps of developing the mobile app Tooth ARcademy. The first step was the selection of histology glass slides, followed by digitization, curation, and annotation. A series of multiple-choice questions was prepared, followed by the final incorporation of the resources into the mobile app. **(C)** Whole-slide scanning was performed using Z-stacking technology on an Aperio Digital Slide Scanner (Leica Biosystems). Z-stacking technology takes multiple images at different focal distances and combines them to make a composite image. This composite nature of the digital scans allowed the creation of multiple images from a single digital tissue section.

The work was undertaken between April 2024 and April 2025 at the Mike Petryk School of Dentistry, University of Alberta, Canada. The School has a collection of tissue sections traditionally used for teaching histology. Hundreds of glass slides were screened by a content expert under a light microscope to identify 76 high-quality and diverse tissue sections that matched the curriculum and learning outcomes of the DDS and DH programs. The steps of developing the mobile app are outlined in Fig 1A, and 1B.

### Digitization

Whole-slide scanning was performed on the selected glass slides with 20X and 40X magnification, using an Aperio Digital Slide Scanner (Leica Biosystems) (Fig 1C). Z-stacking technology was employed in the scan, which captures multiple images at varying focal distances and combines them into a composite image [14].

### Curation

The digital files of the histology images were further screened for quality using Aperio ImageScope 12.4.6. High-resolution screenshots of the digital slides of 20X and 40X magnifications were saved in JPEG format and incorporated into the mobile app. The composite nature of the slides allowed the creation of multiple images from a single tissue section (Fig 1C). The images were sorted into three categories:

(i) Tooth: This category includes ground and demineralized longitudinal, cross, and oblique sections of the entire tooth, the crown, and root.
(ii) Soft tissue: This broad group contains major and minor salivary glands, the tongue, and other parts of the oral mucosa.
(iii) Development: This group encompasses the development of teeth in the bud, cap, and bell stages, as well as the eruption of teeth, various stages of embryo development, and the formation of oral structures.

### Annotation

The images of tissue sections were annotated by a content expert. The annotations included a brief description of the type of tissue (e.g., tongue, gingiva) and type of section (e.g., cross, longitudinal, ground), and identification of the major underlying structures (e.g., dentin, enamel).

### Question preparation

171multiple-choice questions were made to test students’ knowledge of oral histology. The questions were image-based, asking for the identification of structural components in a given histological section.

### Incorporation into the mobile app

Both annotated and non-annotated versions of images, along with the questions, were incorporated into the mobile app, Tooth ARcademy. After coding, the app was tested for functionality and accuracy. The compatibility of the app across different devices and operating systems was also tested before it was released to the Apple App Store and Google Play Store. The steps of app development are outlined in Fig 1. Ethics approval is not required for this kind of study. No human participants were recruited in the study.

## Results

### User interface and functionality of Tooth ARcademy

**Fig 2:**
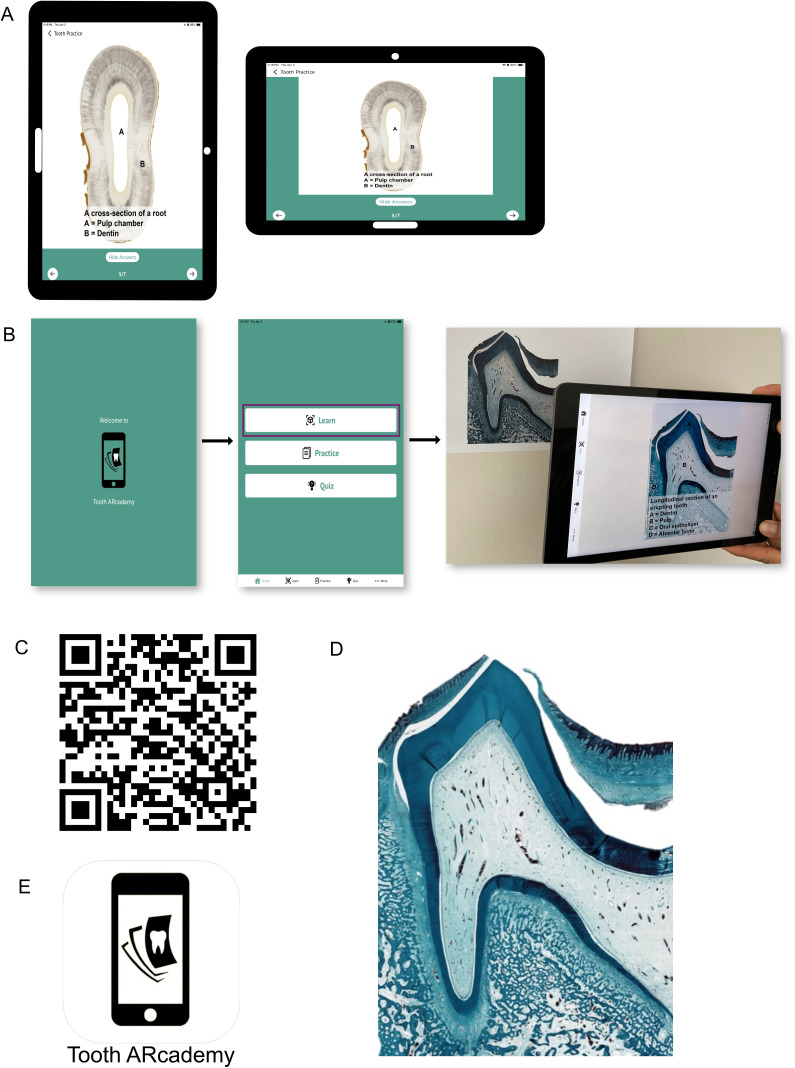
An overview of Tooth ARcademy. (A) Tooth ARcademy is designed to suit various displays. (B) Tooth ARcademy is a mobile app with three modes, *Learn, Practice*, and *Quiz*, which are presented to the user on the homepage. The *Learn* mode is AR-based. When a user views an unannotated target histological section through the device’s camera in *Learn* mode, they can see the annotation for that image. (C) The QR code to download Tooth ARcademy from the Apple App Store. (D) A sample target image to test the AR function of Tooth ARcademy. (E) The logo of Tooth ARcademy.

Tooth ARcademy is designed to provide an easy-to-navigate and interactive user platform, suitable for various devices and displays (Fig 2A). Users can download the app from the Google Play Store or the App Store free of cost. No login or registration is required. The app has three modes, *Learn, Practice*, and *Quiz*, which are presented to the user on the home page (Fig 2B).

#### Learn.

The AR-based *Learn* mode is designed for instructors to facilitate interactive teaching in the classroom and lab. When a user taps the *Learn* button on the home screen, the app requests permission to access the device’s camera. In this mode, when users view an unannotated histological section through their device’s camera, the corresponding annotations appear superimposed on the real image (Fig 2B–2E).

#### Practice.

**Fig 3:**
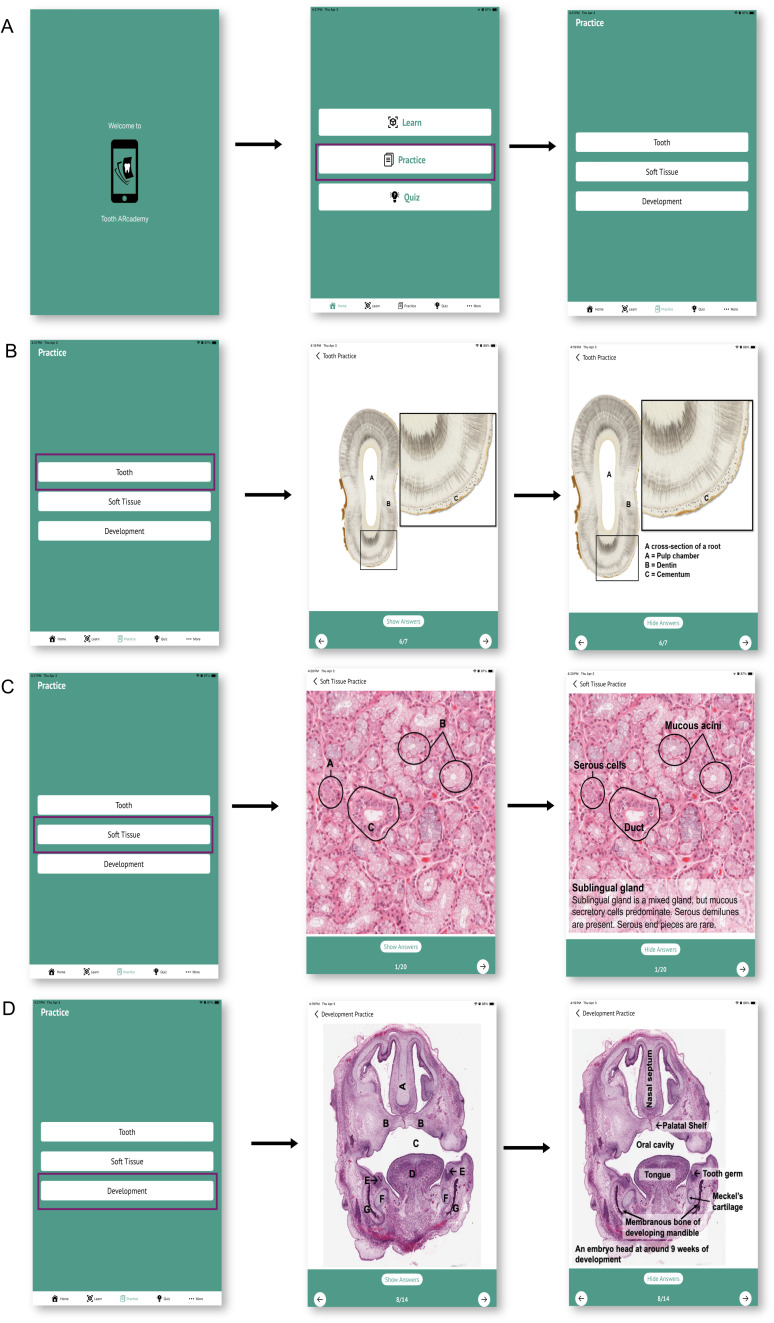
The *Practice* mode of Tooth ARcademy. **(A)** Tooth ARcademy has three modes, Learn, Practice, and Quiz, which are presented to the user on the homepage. **(B)** In the *Practice* mode, students can choose to study from three groups of tissue sections: Tooth, Soft tissue, and Development **(B-D)**. For each category, students are first presented with a histological section, followed by the fully annotated version of the same slide.

The *Practice* mode enables students to reinforce their learning of histology. In this mode, students can choose to study from three groups of tissue sections: Tooth, Soft tissue, and Development. For each category, students are first presented with a histological section, with label lines only, followed by the fully annotated version of the same slide (Fig 3).

#### Quiz.

**Fig 4:**
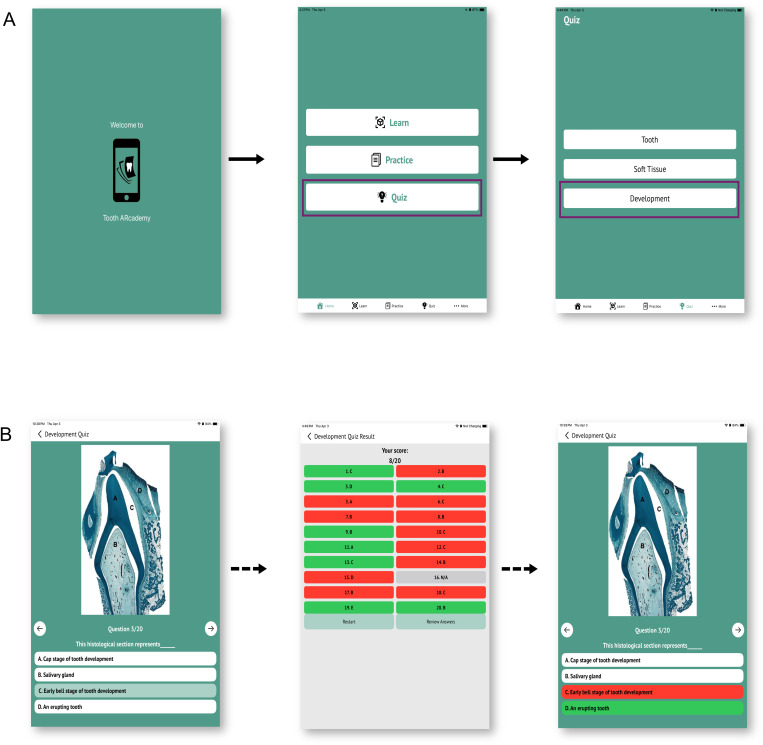
The *Quiz* mode of Tooth ARcademy. **(A)** Tooth ARcademy is a mobile app with three modes, *Learn, Practice*, and *Quiz*, which are presented to the user on the homepage. In the *Quiz* mode, students can choose to study from three groups of tissue sections: Tooth, Soft tissue, and Development. In this mode, students can participate in quizzes that assess their ability to identify and/or label histological images. **(B)** At the end of the quiz, students get their total score and can review their performance on each question.

In *Quiz* mode, students can self-assess their knowledge of histology by participating in quizzes that test their ability to identify and label histological images. Every time a user attempts to take a quiz on a selected topic, the app randomly generates a quiz of 20 questions, drawing from the set of questions of the respective group. At the end of the quiz, students get their total score and can review their performance on each question. When students review their attempts at the end of the quiz, the correct answer is also revealed to them (Fig 4).

### Content of Tooth ARcademy

Tooth ARcademy is programmed to detect 45 target images in the *Learn* mode. The *Practice* section contains 41 images (7 from tooth development, 20 from soft tissues, and 14 from tooth and facial development). Instructors can use some unannotated versions of the images used in the *Practice* mode as target images for the *Learn* mode. The *Quiz* mode contains 171 questions, of which 22 pertain to tooth microanatomy, 81 to soft tissue, and 68 to tooth and facial development.

## Discussion

Knowledge of microscopic structures is essential for the education of dental, medical, and other healthcare professionals. Repeated practice and exposure to a large number of diverse tissue samples are beneficial for students. However, traditional histology teaching in the classroom heavily relies on textbook images and digital slides, where time and the diversity of slides are constraints. Many educational institutes offer supplementary lab sessions for histology. However, such lab sessions are often designed for students to share a limited number of microscopes and glass slides [15]. Tooth ARcademy can serve as a supplementary learning tool for students, allowing them to access a large number of histological sections outside class time. The histological images in Tooth ARcademy are from digital scans of glass slides that were hand-prepared for undergraduate lab teaching under a light microscope. While selecting slides for the app, multiple preparations of the same tissue section were included to represent diversity and common variations that can happen during slide preparation.

The development and incorporation of a mobile app, like Tooth ARcademy, is supported by cognitive load theory, which explains how learning occurs [16]. The knowledge we accumulate throughout our lifetime is categorized as either primary or secondary [17]. While we are evolutionarily programmed to learn primary knowledge, such as speaking, quite easily without assistance, acquiring secondary information requires conscious effort from the learner and is enhanced by explicit instruction [18]. The knowledge of histological structures is an example of secondary information.

Memory formation is an essential part of learning. According to the cognitive load theory, our cognitive system has a working memory with a limited capacity [18,19]. The learning load of working memory is affected by the intrinsic and extraneous loads. The intrinsic load represents the underlying nature of the subject and is not easily changed. The extraneous load arises from how information is presented and can be reduced by altering the way the subject is offered [16,17]. Traditional histology instruction typically combines classroom lectures with hands-on lab sessions using light or virtual microscopes. Students are required to develop a mental model integrating the location, orientation, structure, and function of microscopic tissue components, an effort that imposes a significant cognitive load. Tooth ARcademy can complement traditional teaching methods and alleviate the extraneous load by providing an alternative scaffold for learning histology. The app’s availability on mobile devices provides a key advantage, enabling students to practice anytime and anywhere at their convenience.

Photomicrographs of tissue sections from textbooks typically represent near-perfect slide preparation, rather than common human errors that can occur during slide preparation. Health professionals must differentiate between artifacts caused during slide preparation by human error and those resulting from a pathological condition or developmental anomaly. Exposure to a diverse set of slide collections through Tooth ARcademy can familiarize students with different types of staining (variation in the intensity of colours) and artifacts commonly caused during slide preparation.

The AR-based *Learn* mode of Tooth ARcademy provides opportunities for instructors to innovate in classroom teaching. In this mode, the device camera can be used to reveal the annotation of a target image. The unannotated image collection of Tooth ARcademy is designed to be detected by the device’s camera in AR-based *Learn* mode. Instructors can use screenshots or printouts of the target images to create innovative and playful activities for students. For example, the target images can be used to create escape room games, where revealing the annotation of a specific target image helps students answer questions correctly to unlock a new path in the game. Instructors can use the *Learn* mode of Tooth ARcademy to create a variety of fun in-class activities, such as rapid-fire quizzes or Jeopardy games. The sets of unannotated target images can be used to create additional practice questions for students, allowing them to self-assess their learning outside of the classroom using the *Learn* mode of Tooth ARcademy.

Tooth ARcademy does not need an active internet connection for users to use the app. This feature gives students the freedom to use the app at any time and from any location of their choice. The *Quiz* mode of Tooth ARcademy allows students to independently assess their learning of oral histology. Randomly generated quizzes provide students with a large number of questions, aiming to test their ability to recognize histological sections and their structural components.

In summary, some major strengths of innovations like Tooth ARcademy in education include the technology’s ability to immerse students in their learning. The app provides high-quality, curated content that would otherwise be difficult for students to access, and the integrated learning tools facilitate active recall and formative self-assessment.

### Limitations

At this stage, the image collection of Tooth ARcademy aims to meet the learning outcomes of the first-year DDS and DH students at the Mike Petryk School of Dentistry. Another limitation of the current app is that it does not display the progress of students’ performance over time and does not save records of students’ previous quiz performance. A progress report is beneficial for students to stay motivated, identify learning gaps, and adjust learning strategies accordingly. We acknowledge that data exploring students’ perceptions of using the app is not included in this study.

### Future plans

In the future, we plan to expand the data collection of the app by including diverse images from other parts of the body and explore students’ perceptions of using the app as a learning tool. Interactive features such as drag-and-drop labeling of histological sections will be added to the app, allowing students to label a given histological section. Besides MCQ, other types of questions will be added. We also plan to make the *Quiz* mode more challenging for students by introducing time constraints, where students must complete the quiz within a specified timeframe. To further enhance the learning experience, we would like to introduce adaptive difficulty levels to personalize the quiz experience based on the learner’s proficiency, and display their performance in quizzes over time. We believe Tooth ARcademy will be a valuable m-learning tool for dental, medical, and other health professional students.

## Conclusion

The primary goal of this work was to document the design process, pedagogical framework, and technical development decisions to support other educators and developers who wish to integrate AR into their own instructional contexts. Histology is known to be one of the least engaging topics for health professional students [20]. The incorporation of cellphone apps like Tooth ARcademy can help create interactive and engaging learning environments in the classroom.
